# Bilirubin and Redox Stress in Age-Related Brain Diseases

**DOI:** 10.3390/antiox12081525

**Published:** 2023-07-29

**Authors:** John Paul Llido, Sri Jayanti, Claudio Tiribelli, Silvia Gazzin

**Affiliations:** 1 Liver Brain Unit “Rita Moretti”, Italian Liver Foundation, Bldg. Q, AREA Science Park, Basovizza, 34149 Trieste, Italy; johnpaul.llido@fegato.it (J.P.L.); or srij001@brin.go.id (S.J.); silvia.gazzin@fegato.it (S.G.); 2 Department of Science and Technology, Philippine Council for Health Research and Development, Bicutan, Taguig City 1631, Philippines; 3 Department of Life Sciences, University of Trieste, 34139 Trieste, Italy; 4 Eijkman Research Centre for Molecular Biology, Research Organization for Health, National Research and Innovation Agency, Cibinong 16911, Indonesia

**Keywords:** Alzheimer’s disease, dementia, sclerosis, schizophrenia, ataxia, therapy, brain cancers, NRF2, AHR, heme oxygenase

## Abstract

Cellular redox status has a crucial role in brain physiology, as well as in pathologic conditions. Physiologic senescence, by dysregulating cellular redox homeostasis and decreasing antioxidant defenses, enhances the central nervous system’s susceptibility to diseases. The reduction of free radical accumulation through lifestyle changes, and the supplementation of antioxidants as a prophylactic and therapeutic approach to increase brain health, are strongly suggested. Bilirubin is a powerful endogenous antioxidant, with more and more recognized roles as a biomarker of disease resistance, a predictor of all-cause mortality, and a molecule that may promote health in adults. The alteration of the expression and activity of the enzymes involved in bilirubin production, as well as an altered blood bilirubin level, are often reported in neurologic conditions and neurodegenerative diseases (together denoted NCDs) in aging. These changes may predict or contribute both positively and negatively to the diseases. Understanding the role of bilirubin in the onset and progression of NCDs will be functional to consider the benefits vs. the drawbacks and to hypothesize the best strategies for its manipulation for therapeutic purposes.

## 1. Introduction

The redox status of the brain plays a relevant role in brain physiology and functioning, from regulating post-natal central nervous system (CNS) development to contributing to the diseases characterizing aging (e.g., Alzheimer’s disease, Parkinson’s disease, dementia, ataxia, tumors). Both direct oxidative damage and redox-induced alterations of cellular signaling (most of them shared with bilirubin and its metabolic enzymes) have been documented in the so-called physiologic senescence, when a reduction in the antioxidant capability and an increase in mitochondrial dysfunction, cytochrome C release, genome instability, and telomere attrition, are reported and contribute to the onset and progression of neurologic conditions and neurodegenerative diseases (NCDs) [1,2,3,4,5].

**Figure 1:**
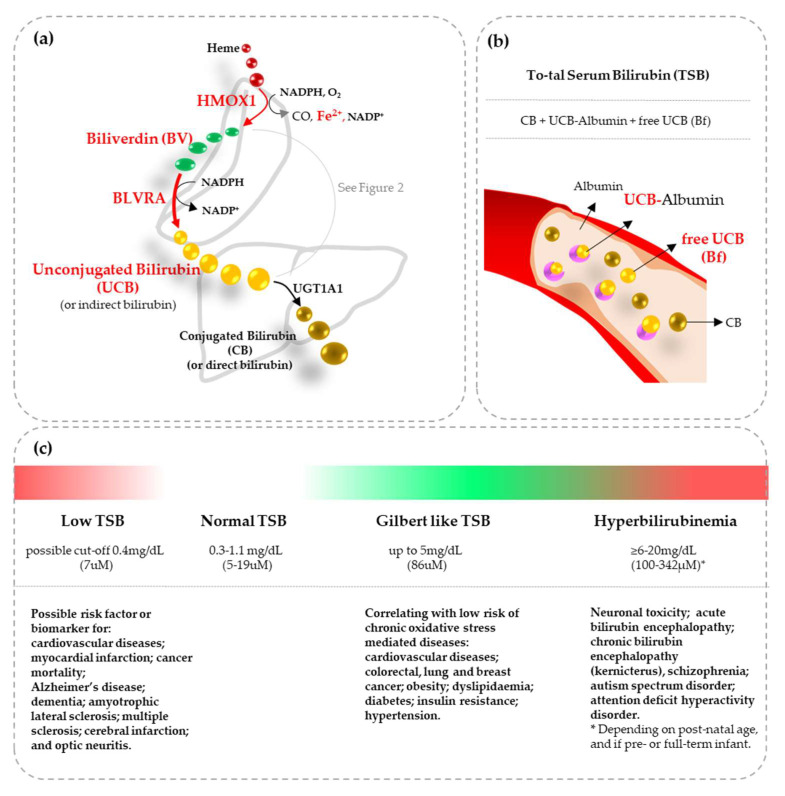
Bilirubin metabolism and clinical correlations among the blood bilirubin level and diseases. (**a**) Systemic bilirubin metabolism. Heme is oxidized to biliverdin (BV), a hydrophilic molecule, by the action of heme-oxygenase 1 (HMOX1), with the concomitant production of CO and Fe^2+^. BV is then converted to bilirubin (at this point unconjugated bilirubin—UCB) by the action of biliverdin reductase A (BLVRA). UCB is highly hydrophobic and requires albumin to stay in solution in the blood and reach the liver. The liver is the deputy organ of body bilirubin clearance. In the liver, UCB is bound to glucuronic acid, becoming conjugated bilirubin (CB), by the action of the uridine diphosphate glucuronosyltransferase (UGT) 1A1 enzyme, making back CB hydrophilic, and easily excreted out the body in form of uro- and stercobilinogen. In red the most relevant to the review intermediated/enzymes. (**b**) The bilirubin species present in blood. Total serum bilirubin (TSB) accounts for UCB bound to albumin (or indirect bilirubin), CB (or direct bilirubin), and a minor part of UCB not bound to albumin (free bilirubin: Bf). Bf is the only circulating bilirubin that can diffuse across cellular bilayers and the blood-brain barrier entering the brain and the cells. When not differently stated, in this paper bilirubin means UCB, and we will focus only on the condition affecting it. (**c**) A resume of the correlation between TSB and chronic oxidative diseases (for references see text). Notably, the review focus on the changes in TSB due to UCB alterations. Reference on panel (**c**) “Low TSB”: 9–12, 23, 48, 72, 176; “Gilbert like TSB”: 9–13, 23, 48, 136, 145, 152, 153, 177, 220; and “Hyperbilirubinemia”: 10, 17, 18, 20, 49, 50, 185–189, 193, 196, 197.

Bilirubin, a heme metabolite (Figure 1a), is not only a recognized powerful cellular antioxidant [6,7,8] but its level has been repeatedly correlated with the risk of developing chronic diseases typical of adult and eldered life (Figure 1c). Bilirubin is now considered as a biomarker of disease resistance, a predictor of all-cause mortality, and a molecule that may promote health in adults [9,10,11,12]. A large body of evidence comes from epidemiologic studies. A low total serum bilirubin (TSB) level (Figure 1b), with a possible cut-off of 0.4 mg/dL (0.7 uM), has emerged as a possible risk factor or as a biomarker for diseases characterized by oxidative impairment. Among them, the list of neurologic and neurodegenerative diseases is long [11,12] (Figure 1c). The moderately elevated TSB present in Gilbert syndrome (GS) subjects, a population with genomic variants partly reducing the activity of the hepatic uridine diphosphate glucuronosyltransferase (UGT) 1A1 enzyme responsible for bilirubin conjugation (Figure 1a), has been correlated with a reduced prevalence of cardiovascular diseases (CVD), diabetes, metabolic syndrome, and certain cancers (Figure 1c) [9,10,11]. In agreement, GS subjects have increased antioxidant status (FRAP—ferric reducing ability potential), as well as reduced pro-oxidant and pro-inflammatory markers (apolipoprotein B—ApoB; C-reactive protein—CRP; interleukin—IL6; and IL1β) than normobilirubinemic subjects. In addition, a stronger protective effect in the fourth to the sixth decade of life was demonstrated [13]. Similarly, it has been suggested that a mild increase of TSB in neonates—the so-called physiologic hyperbilirubinemia leading to jaundice in 60–80% of newborns—may protect them from the oxidative challenge due to birth. In agreement, phototherapy, used to decrease the TSB level in severely hyperbilirubinemic neonates, has been reported to reduce the blood antioxidant capability [14,15,16]. On the contrary, a further increase in TSB may be toxic to the brain [17,18,19,20] (Figure 1c).

**Figure 2:**
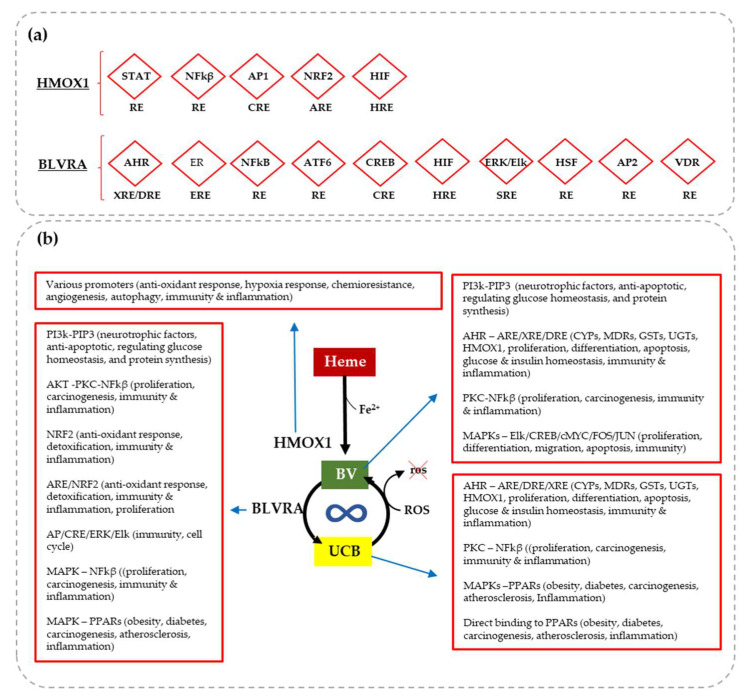
The yellow players (YPs) and their known biologic functions. (**a**) Both HMOX1 (heme oxygenase 1) and BLVRA (biliverdin reductase A) may react on demand under stressor thanks to the multiple binding sites for nuclear transcription factors on their promoter region. StRE: stress-responsive elements; STAT: signal transducer and activator of transcription; NFkβ: nuclear factor-kappa-B; RE: responsive element; AP1/2: AP2: activating enhancer binding protein; CRE: cAMP response elements; NRF2: NF-E2–related factor 2; ARE: antioxidant response elements; HIF: hypoxia-inducible factor; HRE: Hypoxia-responsive element; AHR: aryl hydrocarbon receptor; XRE/DRE: xenobiotic responsive element/DNA replication-related element; ER: estrogen receptor; ERE: estrogen responsive element; ATF6: activating transcription factor6; CREB: cyclic AMP response element binding protein; Elk/ERK: a member of ETS oncogene family/extracellular signal-regulated kinase; SRE: Serum Response Element; HSF: heat-shock factor; VDR: vitamin D receptor. (**b**) In turn, the YPs modulate numerous signaling pathways and transcription factors with potential effects on a plethora of biological functions relevant to the brain and to redox homeostasis. Heme: hemoglobin; BV: biliverdin; UCB: unconjugated bilirubin; ROS: reactive oxygen species (for references see text).

In addition to the “yin” and “yang” properties of serum bilirubin concentration, the presence of the enzymes involved in bilirubin metabolism (the yellow players—YPs, see Figure 2), has been reported in extra-hepatic cells, including the brain. At the cellular level, the YPs will not only produce but also recycle bilirubin. This recycling process accounts for the capability of nM amounts of bilirubin to counteract 10000 times higher amounts of reactive oxygen species (ROS) [6,7,8,21,22]. Additionally, YPs can act on multiple signaling pathways, enter the nucleus, activate the transcription of genes, and act as hormones, amplifying potential biologic functions. Moreover, the promoter of both HMOX1 and BLVRA possesses multiple binding sites for transcription factors (Figure 2a), making them ready to react on demand to stressors and inducers [3,9,11,12,23,24,25,26,27,28,29,30,31,32,33,34,35,36,37,38,39,40,41,42,43,44]. This finding definitively opened a new way of viewing the YPs as homeostatic factors and as part of the cellular defense system [12,23,45].

The YPs contribute to oxidative balance, antioxidant response, detoxification, the lowering of DNA damage and lipid peroxidation, and reducing mitochondrial dysfunction (Figure 2b). Due to bilirubin’s lipophilic nature and its affinity for membrane lipids, a moderate level of bilirubin is known to act as an antioxidant complementary to the cytosolic GSH system in protecting transmembrane proteins and lipids from reactive oxygen species (ROS) and reactive nitrogen species (RNS) attack [7,21], and reducing the platelet prothrombotic phenotype [10] (see also Section 2.6). Moreover, bilirubin blunts inflammation by preserving the mitochondrial transmembrane potential, expressing cytochrome oxidase complex IV, blocking IL1β (interleukin 1 beta) release from macrophages, reducing the level of pro-caspase 1, and inhibiting the NLRP3 inflammasome [46] (see also Section 2.8).

YPs are also involved in the level of neuronal calcium and glutamate balance by inhibiting excitotoxicity and acting on glucose and insulin homeostasis, lipid profile, cell energy, synaptic transmission and voltage control, circadian rhythms, immunity and inflammation, cell cycle, proliferation, differentiation, and apoptosis, and they are anti-microbial and can be used as a pain desensitizer (Figure 2b) [1,3,8,9,11,12,19,23,24,25,26,27,28,29,30,31,32,33,34,35,36,37,38,39,40,41,42,47,48,49,50]. The connections with the senescence of aging are strong. Moreover, in the context of NCDs, the YPs’ antioxidant activity may be of particular importance due to the low intrinsic antioxidant defenses of the CNS [4,51,52,53], combined with the lipid-rich environment requiring high levels of O2, making the brain prone to oxidative stress [2,4,54,55,56,57].

The discussion on the advantages of modulating YPs or delivering exogenous bilirubin to tissues for therapeutic purposes is still ongoing. Of relevance to the discussion, bilirubin has already been used as a constituent on the shells of nanoparticles to exploit its anti-inflammatory and antioxidant properties [3,35,41,42,43,44,51,58,59]. On the other hand, excessive activation of YPs may worsen redox stress, mainly by increasing Fe^2+^ deposition at the site of the lesion and by disrupting the enzymatic activity of BLVRA (see later for details) [3,35,60]. Thus, the use of YPs for therapeutic purposes needs to be further explored and validated.

In this review, we will summarize the potential cross-play between redox imbalance in the age-dependent NCDs and the YPs, and discuss the pros and cons of their therapeutic modulation.

## 2. Oxidative Stress and the Role of Bilirubin in NCDs

### 2.1. Alzheimer’s Disease (AD)

Alzheimer’s disease (AD) is the most frequent neurodegenerative disease that causes gradual cognitive and memory deficits. Brain atrophy, amyloid-β (Aβ) plaques, and neurofibrillary tangles (NTF) in the brain are among the hallmarks of AD [61]. These features of AD are mainly induced by an imbalance between antioxidant defenses and the pro-oxidant status, either due to the increase of a free radical or the lack of antioxidants [62]. Protein carbonyls, lipid peroxidation, and RNA oxidation are increased in the human AD brain [63,64,65]. Oxidative stress is closely linked with pathological events in AD, including mitochondrial dysfunction, impaired calcium homeostasis, metal dysregulation, protein misfolding, impaired autophagy, and inflammation. A single-cell transcriptomic study from an NFT-bearing neuron of the human AD brain revealed that oxidative phosphorylation and mitochondrial dysfunction are highly cell-type-dependent [66]. Oxidative stress leads to metabolic alterations in the AD brain, including glycolysis, calcium regulation, lipid metabolism, mitochondrial processes, and the activation of mTOR (mammalian target of rapamycin) complex 1, which results in reduced autophagy and the emergence of insulin resistance [67].

Both HMOX1 and BLVRA protein levels have been found to increase in the hippocampus of AD and mild-cognitive-impairment subjects. Belonging to the antioxidant cellular defense, their up-regulation has been interpreted as a reaction to the ongoing redox stress. Notably, the activity of BLVRA was decreased. The increased HMOX1 activity, combined with a decreased BLVRA activity, was suggested to lead to the accumulation of CO and iron, enhancing the nitrosative/oxidative stress and inducing changes in the BLVRA protein structure, leading to a non-functional enzyme and finally preventing the conversion of biliverdin into the cytoprotective bilirubin [60,68].

**Figure 3:**
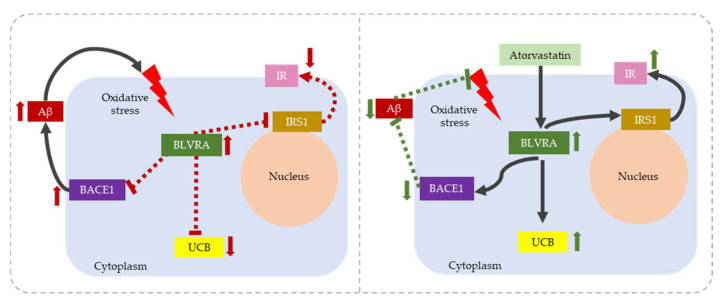
The role of BVRA in BACE-1 mediated insulin resistance in Alzheimer’s disease. Left figure: Oxidative stress promotes BVRA protein modification leading to the increase of its protein level but decreasing its activity including BACE-1 phosphorylation which increases Aβ deposits and UCB production. BVRA impairment also limits IRS1 activation and down-regulates the insulin receptor. Right figures: Atorvastatin target BVRA by restoring its function and mediating the BACE-1 phosphorylation and increasing the production of UCB. Right figure: atorvastatin restores BLVRA function, prevents BACE phosphorylation, decreases Aβ deposits, and up-regulates the IR. Figures are adapted from Triani et al. [69] and Barone et al. [70]. BLVRA: biliverdin reductase A, the adult isoform of the enzyme, BACE1: β-site amyloid precursor protein cleaving enzyme 1, Aβ: amyloid beta, UCB: unconjugated bilirubin IR: insulin receptor, IRS: insulin receptor substrate 1.

Low BLVRA activity has been demonstrated in an in vivo model of AD, where the reduction of BLVRA activity resulted in not only an increase in BACE1 phosphorylation (BACE1: beta-secretase 1, also known as beta-site amyloid precursor protein cleaving enzyme 1), which increased the Aβ deposits, but also in the induction of insulin resistance by down-regulating the insulin receptor (IR) and inhibiting insulin receptor substrate 1 (IRS1—illustrated in Figure 3), supporting the link between insulin resistance and AD pathology [69,70]. The modulation of BLVRA activity in insulin-mediated AD has been evaluated in a canine AD model in which subjects were treated with atorvastatin [69,70]. Atorvastatin increased BLVRA protein level and activity in the parietal cortex, followed by the increase of UCB, with a negative (protective) correlation with oxidative stress markers and cognition [70], confirming the intimate link between AD, redox stress, and the YPs. Also supportive of the molecular mechanisms of action of the YPs are the results obtained in the diet-induced obesity (DIO) mouse model. Bilirubin treatment induced a persistent improvement of insulin sensitivity and an increase in the expression of IR, SREBP1 (sterol regulatory element-binding protein), and PPARγ (peroxisome proliferator-activated receptor gamma) [71]. All of these are known targets of the bilirubin biological functions [11,12], implicating the possibility that UCB counteracts insulin resistance in AD.

In the clinic, TSB levels are reduced in AD patients, as they are in the majority of neurological diseases (Figure 1c) [72]. It is hypothesized that the transition from mild cognitive impairment to obvious AD is aided by reduced UCB concentration [73], pointing to the possible role of UCB in an early event of AD.

In the context of AD, increasing the level of bilirubin in the brain looks protective in three ways: (1) reducing redox stress; (2) reducing Aβ deposits; and (3) reverting insulin resistance.

### 2.2. Parkinson’s Disease

Parkinson’s disease (PD) is the fastest-growing neurological disorder and is characterized by tremors, rigidity, and bradykinesia due to dopaminergic neuron (DOPAn) loss in the substantia nigra. Nonmotor symptoms are also common [74,75]. Among the mechanisms involved in PD, oxidative stress has been recorded in both human samples and experimental models [76,77]. The deficiency and impairment of mitochondrial complex-I activity were found in postmortem studies, and the dopaminergic cell loss induced in animals by toxins and pesticides suggested acting by impairing mitochondrial function [78,79]. Moreover, neuro-inflammation, genetic involvement, and protein disruption that leads to α-synuclein aggregation are listed among the pathologic mechanisms of PD [80].

**Figure 4:**
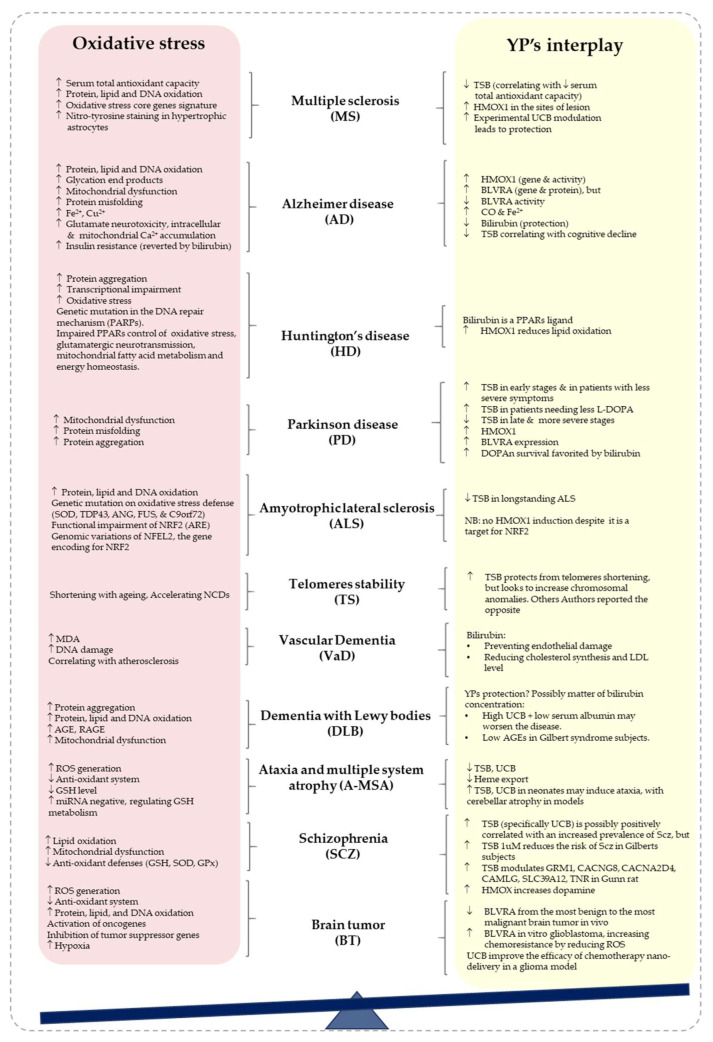
Cartoon summarizing the data on redox stress and YPs interplay in the context of the neurologic conditions. TSB: total serum bilirubin; UCB: unconjugated bilirubin; BVLRA: biliverdin reductase A; DOPAn: dopaminergic neurons; HMOX1: heme oxygenase 1; SOD: superoxide dismutase; TDP43: TAR DNA-binding protein 43; ANG: angiogenin; FUS: FUS RNA binding protein; C9orf72: chromosome 9 open reading frame 72 protein; NRF2: NFE2L2 NFE2 like bZIP transcription factor 2; ARE: antioxidant responsive elements; NFEL2: the gene encoding for the nuclear factor, erythroid 2; PPARs: peroxisome proliferator-activated receptor; AGE: advanced glycation end products; RAGE: receptor for advanced glycation end products; YPs: yellow players; ROS: reactive oxygen species; GSH: reduced glutathione; NCDs: neurologic and neurodegenerative diseases; NMDA: N-methyl-D-aspartate receptor; AHR: aryl hydrocarbon receptor; HMGCR: 3-hydroxy-3-methylglutaryl-CoA reductase; LDL: low density lipoprotein; Gpx: glutathione peroxidase; GRM1: metabotropic glutamate receptor 1; CACNG8: calcium voltage-gated channel auxiliary subunit gamma 8; CACNA2D4: alpha-2/delta voltage-dependent calcium channel; CAMLG: calcium modulating ligand; SLC39A12: solute carrier 39 member 12; TNR: tenascin receptor. References used: Oxidative stress/YP’s interplay. MS, multiple sclerosis: 52, 93–96/97–101. AD, Alzheimer disease: 63–67/60, 68–70, 72, 73. HD, Huntington’s disease: 126–128, 133, 134/12, 135–137, PD, Parkinson’s disease: 76–80/3, 80–83, 85, 86. ALS, amyotrophic lateral sclerosis: 105–107, 110–115, 117–119/120–123. TS, telomere stability: 216, 217/218–220. VaD, vascular dementia: 149–151, 155/9, 136, 152, 153, 156–158. DLB, dementia with Lewy bodies: 139–143/144, 145. A-MSA, ataxia and multiple system atrophy: 201/32, 37, 202. SCZ: schizophrenia: 164–172/173–177, 185–190. BT, brain tumors: 207–209, 212, 213/210, 211, 214, 215.

The hyperactivity of HMOX1 and BLVRA and their potential protection in PD have been reported in detail by Jayanti et al. [80]. In short, the overexpression of HMOX1 has been recorded both in human and PD models [3,81]. PD patients have significantly lower bilirubin/biliverdin ratios than controls, and the ratio is significantly linked with the disease severity [82,83], suggesting a disequilibrium among BV production/conversion to bilirubin and bilirubin production/consumption. TSB has been reported to be higher in the early stages, in patients with less-severe symptoms, and in patients with less need for levodopa (L-Dopa) [80], suggesting an early enhanced antioxidative potential [84]. Moreover, a decrease in TSB accompanies the progression to the late, more-severe stages of PD [80] (Figure 4). Even referring to the state-of-the-art, it is not known if the early increase and then the reduced level of TSB might be explained by the same mechanism of HMOX1/BLVRA activity described previously for AD [60].

Of relevance to the review, in a cellular model of PD, the use of BRUP1, an inducer of bilirubin production via the NRF2-HMOX1 axis (NRF2: nuclear factor erythroid 2-related), was found to counteract rotenone-induced neurotoxicity by suppressing ROS production and protein aggregation [85]. Supportive of this are the results of a recent study using a low-dose of bilirubin, which was able to prevent DOPAn loss in an organotypic brain culture model of the disease [86]. Even though the study found the involvement of oxidative stress in DOPAn demise, as well as the antioxidant activity of bilirubin by modulating the glutathione level, TNFα was demonstrated to be the determinant both in damage and bilirubin-conferred protection [86]. Of importance, redox stress and inflammation are intimately linked and the inhibition of one of them can repress the other [62,87]. Notably, the above-mentioned HMOX1 hyperactivation may enhance ROS production by iron accumulation, a well-known contributor in NCDs [88,89,90]. In this respect, supplying the brain with UCB may be an efficient and safe approach, as demonstrated by Jayanti et al. [86].

### 2.3. Multiple Sclerosis (MS)

Multiple sclerosis (MS) is mainly referred to as a chronic inflammatory immune-mediated disease. MS patients can develop motor, sensory, and cognitive impairments depending on the location of demyelinating lesions [91]. Due to MS’s inflammatory nature, targeting the immune response is the most widely used therapeutic approach but none of the drugs currently used prevents or counteracts progressive neurological deterioration [92].

Several pieces of evidence have linked oxidative damage with MS pathology [93]. Oxidized protein, lipids, and DNA are frequently present in MS plaques and are linked to oligodendrocyte apoptosis and neuronal death in the brains of MS patients [94,95]. In 2020, the relationship between oxidative stress and neuro-inflammation in MS was provided through a single-cell RNA-seq transcriptional profiling strategy in an experimental autoimmune encephalomyelitis (EAE) animal model of the disease. The study revealed that the resident immune cells, as well as the infiltrating monocyte/macrophage clusters, shared an oxidative stress core-gene signature (e.g., CYBB: cytochrome b-245 beta chain; NCF2: neutrophil cytosolic factor 2; NCF4: neutrophil cytosolic factor 4, all of them a sub-unit of the NADPH oxidase; and GPx1: glutathione peroxidase 1) [96] (Figure 4).

Studies on bilirubin protection in EAE started with the induction of HMOX1 and BLVRA, which led to a reduction in the symptoms as well as the inflammatory manifestation in the spinal cords of EAE animals [97,98]. Liu et al. then demonstrated that bilirubin prevents EAE by alleviating oxidative injury [99]. In the clinic, a reduction of TSB is often reported in MS subjects [72]. Notably, MS patients were found to have significantly lower serum total antioxidant capacity, and antioxidant capability is associated with disease severity [100], stressing the potential correlation between bilirubin, the antioxidant defense, and MS. Additionally, higher (more similar to control) TSB was found to be significantly correlated with lower disability status, lower MRI lesions, and shorter disease duration [101]. In the brain, hypertrophic astrocytes and foamy macrophages on the site of the lesion presented an increased nitrotyrosine staining, indicative of redox stress [52,94]. HMOX1 was up-regulated in the microglia—the resident macrophages [94].

Altogether these data show not only a tight link between inflammation and oxidative stress in MS but support the protective role of bilirubin in this condition. Therefore, next to the anti-inflammation and immune-modulator approach, targeting oxidative stress with UCB may provide more benefits. Final data on the pros and cons of the possible alternatives available deserve further, targeted evaluations, to achieve our goal.

### 2.4. Amyotrophic Lateral Sclerosis (ALS)

Amyotrophic lateral sclerosis (ALS) is a motor neuron disease (MND) characterized by progressive and painless muscle weakness due to the degeneration of both upper and lower motor neurons [102]. The disease is rare and is usually diagnosed late because of its clinical presentation heterogeneity and its similarity with other neurological diseases [103]. Death generally occurs 4 years after diagnosis due to respiratory failures [104]. Exposure to environmental factors, including electromagnetic fields, solvents, heavy metals, and pesticides has been linked with enhanced oxidative stress in ALS [105].

Since the life expectancy of ALS patients is relatively short, it is impossible to evaluate oxidative stress biomarkers over a long period. Nevertheless, an increasing number of studies have reported the elevation of protein and lipid oxidation levels, as well as DNA damage from various sample sources, including post-mortem neuronal tissues [106,107] and CSF [108,109], as well as plasma and urine from ALS patients [110]. Indeed, mutations in genes involved in oxidative stress defense have been reported in ALS cases, among them: superoxide dismutase 1 (SOD1), transactive response (TAR)-DNA binding protein (TARDBP, previously called TDP43—possibly by sequestrating miRNA and proteins relevant to mitochondrial functioning and leading to redox stress—[111]), angiogenin (ANG—involved in cell survival against oxidative stress [112,113]), fused-in sarcoma RNA binding protein (FUS—regulating the transcription of oxidative stress protein protection genes [114,115], and chromosome 9 open reading frame 72 (C9ORF72—associated with redox, mitochondrial, and NRF2 pathway imbalance [105,116]). Most of them play their biological action by the transcription factor NRF2. NRF2 modulates redox homeostasis by activating the antioxidant response elements (ARE) system and up-regulating the gene expression of multiple antioxidants, including genes involved in glutathione homeostasis such as cysteine uptake transporter (xCT), glycine uptake transporter (GLY1), and γ-glutamyl-cysteine ligase catalytic and modulatory subunits (γ-GCL-c and γ-GCL-m, respectively) (Figure 4) [29]. For this reason, NRF2 has been considered a therapeutic target both in preclinical and clinical trials of ALS [116]. Nevertheless, the aim of modulating NRF2 as a therapy is hampered by its malfunctioning [117,118]. Genomic variations of NFEL2, the gene encoding for NRF2, have been associated with ALS risks and disease onset [119].

The progressive decrease of TSB level associated with the duration of ALS [120] and the absence of HMOX1 up-regulation despite the induction of NRF2/ARE [121,122] support gene malfunction. Notably, NRF2 is also a major inducer of HMOX1 [29], and the activation of the NRF2/HMOX1 pathway exerts neuroprotection in ALS in vivo models, where the pathway is fully functional [123], supporting the bilirubin-mediated protection in ALS.

The impossibility of modulating HMOX1 as a therapy in the clinic points to the need to deliver bilirubin in the brain in ALS patients. Alternatively, HMOX1 inducers via different signaling pathways (Figure 2) must be developed/used.

### 2.5. Huntington’s Disease (HD)

Huntington’s disease (HD) is an autosomal dominant neurological condition characterized by the expansion of CAG trinucleotide repeat in the gene encoding the huntingtin protein [124,125]. The mutant huntingtin protein disrupts cellular processes, inducing protein aggregation, transcriptional impairment, oxidative stress, and inflammation [126], leading to neurotoxicity (Figure 4). A GWAS study has identified the involvement of genes belonging to the DNA damage-repair system in HD triggered by the presence of ROS [127]. The production of PAR (poly APD-ribose) by PARPs is one of the first steps in the repair of DNA damage. The increase of PARP1 (PAR polymerase-1) and damaged DNA have been detected in the caudate nucleus of severely affected HD brains [128]. Therefore, targeting PARPs is probably one of the promising treatment strategies for HD [129,130,131,132]. PPARα has been identified as a substrate of PARP1 [133]. In addition to controlling energy homeostasis, mitochondrial fatty acid metabolism, excitatory glutamatergic neurotransmission, and also cholinergic/dopaminergic signaling in the brain, PPAR-α also regulates oxidative stress, energy balance, and fatty acid metabolism (Figure 4) [134].

Bilirubin is a direct ligand for PPAR-α, activating its pathway and increasing gene responsiveness [12,135,136]. The induction of HMOX1 has been demonstrated in an in vivo model of HD to be protective by reducing lipid oxidation, inducing antioxidants, and ameliorating inflammation [137], suggesting the protective role of UCB as the end product of HMOX1, through the PARP pathway. Moreover, the pharmacologic modulation of HMOX1 ameliorated the increased lipid peroxidation, nitrite concentration, and decreased endogenous antioxidants, with an improvement of the behavior, especially when combined with lithium chloride administration [137]. Data are still too few to draw a sound conclusion, but an enhancement of YP activity looks to be a promising therapeutic option.

### 2.6. Dementia with Lewy Bodies (DLB)

Dementia with Lewy bodies (DLB) is the second-most-frequent type of dementia after AD, and its typical symptoms include visuoperceptual dementia, which manifests as visual hallucinations, attention disturbances, and Parkinsonism [138]. Amyloid deposition, α-synuclein aggregates, and neuronal loss are among the pathological features of DLB [139,140]. Similar to AD, oxidative stress and mitochondrial dysfunction play a role in DLB pathogenesis. Protein and lipid oxidation as well as DNA damage are significantly increased in the parietal and temporal lobe cortex of DLB patients [141]. Moreover, lipo-oxidation, advanced glycation (AGE), and AGE receptor (RAGE) protein levels are up-regulated in the SN and frontal cortex in the early stages of DLB [142]. Meanwhile, mitochondrial dysfunction is marked by low mitochondrial oxygen uptake and complex I activity in the brain cortex of DLB subjects (Figure 4) [143].

Zhong et al. reported an increased blood level of unconjugated bilirubin and low albumin concentration in DLB patients and suggested a worsening role of bilirubin in dementia as it enhances αβ deposition in the brain; this was supported by their in vitro experiments [144]. On the contrary, Kalousovà recorded low plasma levels of AGEs, particularly pentosidine and nepsilon carboxymethyl lysine, in GS subjects, in agreement with bilirubin-induced antioxidant protection [145]. To the best of our knowledge, data on the prevalence of DLB in the GS population, on HMOX1 and BLVRA expression, or on activity in DLB patients are not available, thus preventing conclusions from being made. Additional research is needed to understand whether the modulation of YPs might be beneficial or not in DLB.

### 2.7. Vascular Dementia (VaD)

Vascular dementia (VaD) is an umbrella term comprising dementia, caused by a large group of heterogeneous vascular brain lesions [146]. Cognitive alterations in VaD are different than in AD and are highly dependent on the neuronal region affected by vascular pathology [147]. VaD can be caused directly by having a stroke due to small vessel diseases such as cerebral micro-bleeds and cerebral micro-infarct, which is associated with atherosclerosis (fatty deposits accumulate to restrict vascular luminal diameter) formation [148]. The involvement of oxidative stress in VaD has been marked by the increase of malonaldehyde (MDA, a lipid peroxidase marker) and the oxidative DNA damage repair markers (e.g., SOD, CAT, GPx, GR, and/or oxo-guanine) in CSF and urine of VaD subjects (Figure 4) [149,150]. A 20-year prospective study revealed the association between atherosclerosis with the development of VaD [151].

Even if studies on the correlations between VaD and YPs are not available, bilirubin is known to be an anti-atherogenic molecule modifying cholesterol oxidation [152] and inhibiting cholesterol synthesis [153], thus reducing plaque formation [153]. Moreover, the antioxidant activity of bilirubin at the cellular membrane level has been reported to reduce the mean platelet volume and hyper-reactivity, as well as the prothrombotic phenotype, which is of possible relevance to VaD [10]. Bilirubin also affects AHR (aryl hydrocarbon receptor), a ligand-activator transcription factor. AHR is overexpressed in human atherosclerosis vessels [154] and is known to mediate oxidative stress and inflammation in atherosclerosis [155]. Interestingly, the activation of AHR by bilirubin is believed to have an athero-protective effect [9,136,156] via an anti-inflammatory action [157,158]. This confirms the complexity of the AHR pathway and supports the anti-atherogenic roles of bilirubin in VaD. Notably, due to the systemic nature of VaD, the increase of TSB might be a practicable approach.

### 2.8. Schizophrenia (Scz)

Schizophrenia (Scz) is a serious mental disorder characterized by the impaired perception of reality and corresponding changes in behavior [159,160]. Symptoms include psychosis (hallucinations, delusions, thought disorder, movement disorder) and negativity (loss of motivation, loss of interest or enjoyment in daily activities, withdrawal from social life), and are cognitive (attention, concentration, and memory) [161,162,163].

The role of oxidative stress in schizophrenia has been well-described, and several pathophysiological mechanisms including lipid peroxidation-induced neuronal damage, reduced antioxidant defenses, redox dysregulation of transcriptional factors, noncoding RNAs, epigenetic mechanisms, mitochondrial dysfunction, neurotransmitter alterations (e.g., glutamate, dopamine), metabolic abnormalities, and genetic variants associated with the previous mechanisms (e.g., on GSTs; glutathione cysteine ligase –GCL; Cacna1c—calcium voltage-gated channel subunit alpha1 C) have been documented (Figure 4) [164,165,166,167,168,169,170,171,172].

The correlation between TSB and Scz is debated [173]. Becklén et al. found a reduced TSB level in first-episode psychosis Scz patients (FEP, average 0.64 mg/dL) with respect to healthy subjects (average 0.88 mg/dL). The lower TSB was associated with a longer duration of the untreated psychotic crisis. The author hypothesizes that the decreased bilirubin level resulted in reduced antioxidant protection [174]. Further supporting the role of bilirubin in Scz is the observation of a higher level of the pigment in patients in relapse (0.38 mg/dL) versus partial remission (0.34 mg/dL) [175]. Other studies reported a higher frequency of Scz in GS subjects where TSB is higher, suggesting either that a higher concentration of the pigment is a cofactor of the diseases or the existence of a genetic sub-type of Scz [176]. The last hypothesis might explain the opposite results obtained by Vitek et al., who reported in GS subjects a lower risk of Scz by up to 19% [177]. The causal connection between the pigment and Scz is not understood, and more studies are required. Of relevance, at high levels, bilirubin is known to bind to cellular membranes and modify their fluidity, interfering with mitochondrial cellular respiration and inducing a cellular energetic crisis and the production of ROS [178,179,180]. Moreover, highly elevated bilirubin activates microglia and the NLRP3 inflammasome and induces the release of pro-inflammatory molecules and glutamate, causing calcium imbalance, affecting neurotransmission, cellular division and differentiation, migration and myelination [10,49,181]. Furthermore, low physiologic concentrations of bilirubin have been reported to inhibit the inflammasome and related cytokine release in in vitro experiments on macrophages [182]. The relevance to microglia, the CNS resident macrophages, is not known. Notably, Scz is also considered a neurodevelopmental disease. In the neurodevelopmental hypothesis of Scz, perinatal challenges alter the synaptic formation and/or functioning, leading to manifestations at an adult age [183,184]. It is noteworthy to mention that the frequency of Scz is higher in subjects experiencing neonatal hyperbilirubinemia [185,186,187,188,189]. Alterations of schizophrenia-associated genes (GRM1—metabotropic glutamate receptor 1; CACNG8—calcium voltage-gated channel auxiliary subunit gamma 8; CACNA2D4—voltage-dependent calcium channel complex alpha-2/delta subunit; CAMLG—calcium modulating ligand; SLC39A12—solute carrier family 39 member 12; and TNR—tenascin R) were reported in the Gunn rat [190]. All of them are involved in the formation of the synaptic circuits, supporting the concept of Scz as neurodevelopmental disorder [191,192,193]. Moreover, most of these genes belong to the glutamate system, with glutamate perturbation being a shared characteristic of Scz and bilirubin-induced neurotoxicity [194,195,196,197]. This supports the interplay of excitotoxicity, calcium, and mitochondria in the triad in synaptic neurodegeneration [198].

In conclusion, the interplay of Scz and bilirubin is still confusing due to the lack of relevant information on the threshold of bilirubin differentiating protection from toxicity. Focused studies are needed to unravel the role of YPs in Scz and suggest their therapeutic application.

### 2.9. Ataxia and Multiple System Atrophy (A-MSA)

Ataxia (A-) is a group of symptoms of poor muscle control that usually results from cerebellar or brainstem damage, which can cause problems with walking, balance, coordination, speech, and eye movements [199,200]. Although the causes, onset, and progression of ataxia remain to be fully understood, oxidative stress via the excess generation of ROS and/or disruption of the antioxidant system results in cellular damage (Figure 4) [201]. It has to be mentioned that HMOX1 hyper-activation and Fe^2+^ accumulation have been reported to contribute to specific forms of ataxia, namely Friedreich and posterior column sensory ataxia [32,37]. TSB, namely UCB, was significantly lower, while homocysteine level, an oxidant biomarker, was higher in multiple system atrophy (MSA) patients than in healthy controls [202]. These data are essential because they simultaneously support the inverse correlation between TSB and a pro-oxidant status (homocysteine) and support bilirubin as a systemic antioxidant effector. In addition, the negative correlation between a low UCB and the presence of MSA indicates that bilirubin is a predictor, risk factor, or marker for developing the disease.

Solid clinic and research data are still needed, but the preliminary information suggests that the supplementation of bilirubin appears to be a safe and indiscriminate approach to treating A-MSA.

### 2.10. Brain Tumors in the Elderly

Brain tumorigenesis is favored in the elderly population by a progressive decrease of the antioxidant mechanisms, increasing the susceptibility to developing genetic mutations, oncogene activation, loss of tumor-suppressor gene function, angiogenesis, and a micro-tumor environment [203,204,205,206] (Figure 4).

The malignancy and response to treatments strongly depend on the delicate equilibrium between the oxidant and antioxidant environment of the tumor [207,208,209]. Meningiomas and low- and high-grade gliomas—the two most common brain cancers—present an increased protein and lipid peroxidation level and a decreased antioxidant capacity compared to control samples.

The BLVRA level was reduced, with a decreasing trend from the most-benign to the most-malignant brain tumors, in agreement with the increased pro-oxidant status of the cancer cells. The correlational studies among BLVRA expression and redox markers revealed a negative correlation with the advanced oxidation protein products; but a positive correlation with the ferric-reducing antioxidant power [210]. Due to the well-known antioxidant actions of the YPs, BLVRA down-regulation in brain tumors has been suggested as a biomarker [210]. Moreover, the induction of a hypoxia-mediated pro-oxidant environment in a human glioblastoma cell line was reported to enhance chemoresistance. BLVRA level was also up-regulated. Depletion of the BLVRA gene enhanced chemoresistance and intracellular ROS levels, supporting the protective role of the YP’s induction as an antioxidant [211]. Chemotherapy efficacy is also limited by the low brain/tumor accessibility due to multidrug resistance transporters (MDRs), Pgp (P-glycoprotein), and BCRP (breast cancer resistance protein) expressed at the blood–brain barrier and in glioblastoma cells [212,213]. Both transporters are up-regulated by bilirubin [214,215].

Further information is needed before we are able to balance the two opposite effects. The successful delivery of UCB pegylated nanoparticles loaded with chemotherapeutic agents to glioma in mice offers preliminary data on a promising approach [51].

### 2.11. Telomere Stability in Neurodegeneration

Aging can also be considered as the accumulation of senescent cells contributing to body dysfunction. Telomeres—the genomic portions of TTAGGG repeats located at the ends of linear chromosomes—are bound and protected by a sheltering protein complex from being recognized as DNA damage that triggers a DNA damage response. Standard DNA polymerases replicate DNA templates without telomerase and nucleolytic processing generates chromosomes with progressively shortened telomeres after DNA replication. Beyond the critical length and stability, telomeres bind fewer capping proteins and are sensed as exposed DNA ends and misinterpreted as DNA damage, resulting in cell cycle inhibition and arrested proliferation. Telomere dysfunction, together with mitochondrial dysfunction (another hallmark of ageing) and oxidative stress, may accelerate the progression of neurodegenerative disorders like PD and AD [216,217] (Figure 4).

A significant positive association exists between mildly increased TSB and telomere length (TL) [218], similar to the longer telomeres observed in male individuals with mildly elevated bilirubin (GS), as well as in Gunn rats [219]. While a decrease of the nuclear anomalies reflecting DNA instability were reported in older individuals with GS, suggesting a protective effect of bilirubin against the consequence of variation in the genetic material [220], the opposite (increased chromosomal anomalies such as nucleoplasmatic bridges and nuclear buds) were observed in an animal model [219].

The discrepancy may be due to the bilirubin level, the duration of the challenge, or the model per se (human vs. rat).

## 3. Bilirubin as a Therapy

As we have discussed the potential of bilirubin’s protective role in oxidative stress-mediated neurological diseases (Figure 4 and Table 1), the next question should be how we modulate bilirubin to reach a neuroprotective concentration.

The first way is by inducing the systemic concentration of total serum bilirubin by HMOX1 inducers [41,107,221,222,223]. HMOX1 is easily induced by clinically used drugs with a recorded increase of TSB (e.g., some NSAIDs and hypolipidemic agents) [58]. This method will unfortunately provide no control regarding the amount of UCB that can enter the brain.

The second option is to enhance the UCB production in the brain by inducing HMOX1 locally. However, the activation of HMOX1 is followed by the increase of its side products, including iron, which will add more harm to already pre-existing HMOX1 hyper-activation in several neurological diseases [3,35,72].

The third option is to deliver the desired amount of UCB into the brain and, even better, into the targeted region. It is not an easy task, and the use of nanoparticles may be a solution [224,225,226]. The use of bilirubin nanoparticles has been studied in glioma in mice [51]. Interestingly, the future application of advanced technology such as the magnetic nanoparticle can be a sophisticated tool given the possibility to control the delivery in a paramagnetic field [227,228,229]. Further studies are needed to reach the use of UCB at this point.

## 4. Conclusions

A considerable amount of evidence suggests that bilirubin might be beneficial to the redox imbalance ongoing in aging. This supports the idea that increasing its level could be a prophylactic or therapeutic approach, although the modulation of YPs in the brain might also results in side effects. We still have a long way to go, but it is indubitable that bilirubin looks a very promising natural compound for use in the treatment of several NCDs.

## Figures and Tables

**Table 1:** The main biomolecular effects of the YPs.

	Heme	HMOX	Fe^2+^	BV	BLVR	UCB
Changes During Disease	Accumulating in the site of lesion	Usually induced (chemical induction, inhibition, and Ko models frequently used to assess its biologic and pathologic functions)	Increased as part of BBB breakdown, hemorrhage and HMXO1 induction.	Rarely quantified. Suddenly added to model of diseases to assess its functions.	Usually induced (with possible induction of defects in its enzymatic activity in high redox stress environment). Fewer chemical inducers/inhibitors are available to assess its functions. KO models are seldom used for this purpose.	TSB: both increased and decreased. Supposed to be increased if HMXO and BLVR induced Seldom added to models of diseases to assess its functions.
Target and Effect	Protective Reducing apoptosis and inducing SOD and HMOX1, mitochondrial functions and cytochrome C release, and ferritin production [30,31,137]. Enhancing redox stress and heme release, protein and lipid oxidation, metalloproteinases release and tissue damage, inhibiting the antioxidant response through NRF2, and impairing the proteasome and unfolded protein response, inducing mitochondrial dysfunctions and mitophagy and apoptosis (Frederic ataxia, posterior column ataxia, neurodegenerative diseases) [32,37].	Protective Reducing redox stress, increasing survival, inducing the transcription of the stress response genes, reducing lipid peroxidation [89] and inducing the synthesis and release of GSH [137]. Promoting proliferation and neuronal survival via PI3K/Akt/BDNF signaling, even migrating into the nuclei and acting as a transcription factor [9,11] (AD, PD, ischemia, HD [38]). Improving glutamate neurotoxicity, mitochondrial damage [137]. Antioxidant (by producing BV, UCB, and acting as a transcriptional factor [9,11]). Potentially dangerous if excessively induced (AD, PD, SCZ, Stroke, trauma [3,60]). Increasing cholesterol and products of cholesterol oxidation [99]. Increasing Fe^2+^ production in turn enhancing DNA damage, cell bioenergetic failure, mitophagy and autophagy, oxidizing catecholamine [3,60].	Damaging Worsening redox stress, enhancing protein and lipid oxidation, and DNA damage. Reducing SOD activity, inducing a cell bioenergetic failure, apoptosis, neuronal autophagy, damaging the BBB (via NFkβ, AP1) [32,89].	Protective Levering DNA damage (possibly by scavenging ROS directly or after conversion into UCB [27]), inducing BLVR translocation into nucleus [9,11], with multiple anti-inflammatory actions [9,11].	Protective Protective in meningioma and glioma [220], and EAE [98]. Modulating Tau deposition [43]; enhancing neuronal and synaptic plasticity (MAPK/PI3k) [60], Reducing apoptosis (MAPK/Akt [9,11]) Activating the stress responses gene (including HMOX) [29], ameliorating insulin brain resistance [70]. Inducing chemoresistance [211]. Missed Protection Missed protection in AD (gene up, activity down [60,68,73]).	Protective Protective (EAE, PD, stroke, ischemia, traumatic brain injury, cerebral atherosclerosis, glioma, etc. [11,51,86,99]). Activating the antioxidant response (NRF2 [29]); boosting survival and repair (AKT/CREB/BDNF [9,11]); increasing mitochondrial respiration, AMPA and Ca channels [11]; enhancing the transcription of the detoxification system (CYPs, UGT, by MAPK/NRF2) [11,156], inhibiting NMDA excitotoxicity and related neuronal death [28] Damaging Responsible for acute and chronic bilirubin encephalopathy (kernicterus), and suggested increasing the risk of ADHD, SCZ, autism [194], by inducing a plethora of mechanism (among them oxidative stress, apoptosis, glutamate neurotoxicity, inflammation, epigenetic alterations of brain development, reduced myelinating, cell death, ca imbalance, etc. [49,194]).
